# Altered Amplitude of Low-Frequency Fluctuations and Functional Connectivity in Excessive Daytime Sleepiness in Parkinson Disease

**DOI:** 10.3389/fnins.2020.00029

**Published:** 2020-01-31

**Authors:** Xixi Wang, Min Wang, Yongsheng Yuan, Junyi Li, Yuting Shen, Kezhong Zhang

**Affiliations:** ^1^Department of Neurology, The First Affiliated Hospital of Nanjing Medical University, Nanjing, China; ^2^Department of Neurology, Nanjing First Hospital, Nanjing Medical University, Nanjing, China; ^3^Department of Radiology, The First Affiliated Hospital of Nanjing Medical University, Nanjing, China

**Keywords:** excessive daytime sleepiness, Parkinson disease, resting-state functional MRI, amplitude of low-frequency fluctuations, functional connectivity

## Abstract

**Object:**

Excessive daytime sleepiness (EDS) is common in Parkinson disease (PD), but the neural basis of EDS in PD is unclear. We aim to analyze the neural activity changes in PD-related EDS.

**Methods:**

In the present study, 38 PD patients and 19 healthy controls underwent clinical assessments and resting state functional magnetic resonance imaging (MRI) at 3T. Patients were further classified into PD patients with EDS (*n* = 17) and PD patients without EDS (*n* = 21), according to the Epworth Sleepiness Scale (ESS) cutoff score with greater than 10 or less than 3. We evaluated all patients using PD-related motor and non-motor clinical scales. An analysis of covariance and *post hoc* two-sample *t*-tests were performed to examine between-groups differences of the amplitude of low-frequency fluctuations (ALFF) and functional connectivity (FC).

**Results:**

We found that, all PD-EDS subjects in our study were male. Compared with the control subjects, PD patients with EDS had decreased ALFF in the Pons and increased ALFF in the Frontal_Mid_Orb_L (*p* < 0.01, corrected). Moreover, PD patients with EDS showed decreased ALFF in the left posterior cingulate cortex (PCC) relative to PD without EDS, which was negatively correlated with the ESS score (*p* < 0.001). After that, the FC analysis with the left PCC region of interest showed reduced FC of the right PCC and right precuneus in PD with EDS compared with PD without EDS (*p* < 0.01, corrected).

**Conclusion:**

We hypothesized the wake-promoting pathways and the default mode network dysfunction underlying the EDS in male PD patients.

## Introduction

Excessive daytime sleepiness (EDS) is a common feature of Parkinson disease (PD). It manifests as unintentional or inappropriate sleep, namely, inability to stay awake during the day. A large proportion of patients with PD develop EDS with longer disease duration (Zhu et al., 2016), which contributes to poor quality of patients’ life and increases the risk of harm, such as traffic accidents (Weerkamp et al., 2013). Previous studies found that EDS is a separate manifestation of PD (Hoglund et al., 2015) and supported the hypothesis of EDS representing a form of primary insomnia. During last few decades, focus was drawn to EDS and its correlated factors in PD (Goldman et al., 2013; Zhu et al., 2016); however, current treatment options for EDS in PD are very limited (Rodrigues et al., 2016) and its pathogenesis is poorly understood.

In fact, regarding EDS in PD, several neuroimaging methods have been used to preliminarily explore cerebral changes, including structural magnetic resonance imaging (MRI) (Gama et al., 2010; Kato et al., 2012), diffusion tensor MRI (Matsui et al., 2006b; Chondrogiorgi et al., 2016), single photon emission computed tomography (SPECT) (Matsui et al., 2006a; Happe et al., 2007), and resting state-functional MRI (rs-fMRI) (Wen et al., 2016). Nevertheless, conflicting results existed among those researches due to the lack of healthy controls or the limitations of topical brain analysis.

Rs-fMRI, examining whole-brain spontaneous fluctuations in the blood oxygen level dependent (BOLD) signal of functional MRI without any explicit input or output, indirectly shows a manifestation of spontaneous neuronal activity (Fox and Raichle, 2007). For example, the amplitude of low-frequency fluctuations (ALFF) measures the spontaneous amplitude of low-frequency (0.01–0.08 Hz) BOLD signal at the local level (Yang et al., 2007); whereas, functional connectivity (FC) indicates inter-regional temporal patterns of the BOLD signal at the network level (Fox and Raichle, 2007). In recent years, they have been applied widely to the study of neuropathologic mechanisms (Rosazza and Minati, 2011; Lee et al., 2013).

In the present study, to address the association of EDS in PD patients and neural activity changes, we focused on rs-fMRI combining the ALFF with FC approaches.

## Materials and Methods

### Participants

In total, 64 patients enrolled in the study were required to meet the following criteria: (1) meeting the diagnostic criteria for idiopathic PD according to the United Kingdom Parkinson Disease Society Brain Bank criteria; (2) without medical history of traumatic brain injury stroke, brain tumor, dementia, or psychiatric disorders; (3) without contraindications for MRI scan; (4) a Mini Mental State Examination (MMSE) score greater than 24; (5) not taking sedative and hypnotic medications; and (6) excluding sleep disorders such as insomnia, restless leg syndrome, narcolepsy, and obstructive sleep apnea that may contribute to EDS. Patients were recruited consecutively from the outpatients at our hospital, and were at first clinically evaluated by a neurologist, who is a trained movement disorder specialist and sensitized to psychiatric disorders in PD. Patients were also evaluated about sleep symptoms in a face-to-face interview and by the specific questionnaire, the Epworth Sleepiness Scale (ESS).

The ESS contains eight items with a score ranging from 0 to 24 that measure the subject’s expectation of dozing in eight daily situations within the past 3 months. This questionnaire, with satisfactory clinimetric results (validity, reliability, and sensitivity), is recommended by Movement Disorder Society for rating daytime sleepiness to categorize and measure severity (Hogl et al., 2010). Thus, we classified PD patients into PD patients with EDS (PD-EDS) (*n* = 17) with a ESS score of 10 or greater, and PD patients without EDS (PD-non-EDS) (*n* = 21) with a ESS score 3 or less (Johns, 1991; Hogl et al., 2010; Kato et al., 2012); Meanwhile, patients with ESS scores from 3 to 10 were excluded (*n* = 26).

Subsequently, we evaluated all patients using clinical scales. Motor-related assessments included the unified Parkinson disease rating scale (UPDRS-III) score, the postural instability and gait disorder (PIGD) score, the tremor score, and the Hoehn-Yahr (H-Y) staging score. Cognition-related assessments included the MMSE and the Frontal Assessment Battery (FAB). Other sleep assessments included the Parkinson’s Disease Sleep Scale (PDSS) and the REM Sleep Behavior Disorder Screening Questionnaire (RBDSQ). The Hamilton Anxiety Scale (HAMA) and the Hamilton Depression Scale (HAMD) were used to detect the severity of anxiety and depression separately. In addition, we calculated total levodopa-equivalent daily dose (LEDD), LEDD of levodopa preparations, and LEDD of dopamine receptor agonists of each PD patient according to the previous description (Tomlinson et al., 2010). MRI scans and clinical examinations took place while all patients had been anti-Parkinson free for at least 12 h. In addition, age- and education- matched control subjects (HC) (*n* = 19) were recruited for this study. None of these controls had any history of neurological or psychological disorders. The present study was approved by the ethics committee of our institution. Informed consent was obtained from all individual participants included in the study.

### MRI Acquisition

MRI scanning was performed with a 3.0 T Siemens MAGNETOM Verio whole-body MRI system (Siemens Medical Solutions, Germany) equipped with eight-channel, phase-array head coils. We used tight foam padding to minimize head movement and ear-plugs for reducing noise. Subjects were instructed to remain motionless, think nothing and avoid falling asleep with our reminders. Three-dimensional T1-weighted anatomical images were acquired using the following volumetric 3D magnetization-prepared rapid gradient-echo (MP-RAGE) sequence (repetition time (TR) = 1900 ms, echo time (TE) = 2.95 ms, flip angle (FA) = 9°, slice thickness = 1 mm, slices = 160, field of view (FOV) = 230 × 230 mm^2^, matrix size = 256 × 256 and voxel size = 1 × 1 × 1 mm^3^). Resting-state functional images were collected using an echo-planar imaging (EPI) sequence (TR = 2000 ms, TE = 21 ms, FA = 90°, FOV = 256 × 256 mm^2^, in-plane matrix = 64 × 64, slices = 35, slice thickness = 3 mm, no slice gap, voxel size = 3 × 3 × 3 mm^3^, total 4 volumes = 240).

### Data Processing

The data were analyzed using the data processing assistant for resting-state fMRI (DPARSF^1^) (Chao-Gan and Yu-Feng, 2010) with Statistical Parametric Mapping (SPM8^2^). Steps included: (1) removal of the first 10 time points; (2) slice timing correction; (3) head motion correction via six-parameter rigid body spatial transformation during data acquisition; (4) non-linear registration of the high-resolution T1 structural images to the Montreal Neurological Institute (MNI) template, in which T1 structural images were segmented as white matter, gray matter, and cerebrospinal fluid using a new segment algorithm with DARTEL (diffeomorphic anatomical registration through exponentiated lie algebra), followed by further structural analyses of the resulting segments; (5) nuisance signal removal (white matter, cerebrospinal fluid, global signal, 6-head motion parameters as covariates) via multiple regression; (6) spatial normalization to the MNI template; (7) resampling of images into a spatial resolution of 3 × 3 × 3 mm^3^; and (8) spatial smoothening with a Gaussian kernel (full-width at half-maximum = 4 × 4 × 4 mm^3^). We excluded subjects from further analysis if the translation or rotation of head movement was greater than 2 mm or 2° in any direction.

### ALFF Analysis

The ALFF calculation procedure was as follows: (1) fast Fourier transform (FFT) was used to convert all voxels from the time domain to the frequency domain; (2) the ALFF of every voxel was calculated by averaging the square root of the power spectrum across 0.01 Hz to 0.08 Hz; and (3) the resulting ALFF was converted into z-scores by subtracting the mean and dividing by the global standard deviation for standardization purposes.

### FC Analysis

Before FC analysis, band-pass filtering (0.01 < f < 0.08 Hz) was performed and the linear trend was removed for weakening the linear drift of rs-fMRI signal time series. Then FC analysis was performed using the Resting-State fMRI Data Analysis Toolkit^3^. Based on the ALFF result, the region that showed significant difference between the PD-EDS and PD-non-EDS subject groups and correlation with ESS score was defined as the region of interest (ROI). After that, we performed a voxel-wise FC analysis by computing the temporal correlation between the mean time series of the ROI and the time series of each voxel within the brain. Pearson correlation coefficient maps were created for each individual subject and were converted to a *z*-value using the Fisher z transformation (Wang et al., 2018).

### Statistical Analysis

The clinical data were analyzed using IBM SPSS statistics v20.0.0 software (SPSS, Chicago, IL, United States). We employed Fisher exact test for gender, as well as one-way analysis of variance (ANOVA) for age and gender and two-sample *t* tests for the remaining variables.

An analysis of covariance (ANCOVA) was performed to examine brain areas with significant differences among the three groups with age, gender, education, and gray matter volume as nuisance variables (voxel-level *p* < 0.01, cluster size >22 voxels, corresponding to a corrected *p* < 0.01 as determined by AlphaSim correction)^4^. These areas were then extracted as a mask. Next, we performed two-sample *post hoc t* tests within this mask to further detect significant differences between groups, controlling for the same covariates mentioned previously (voxel-level *p* < 0.01, cluster size >5 voxels, corresponding to a corrected *p* < 0.01 as determined by AlphaSim correction). Subsequently, we extracted the cluster showing significant ALFF difference between PD-EDS and PD-non-EDS groups and calculated the average ALFF value of which to explore the correlation with ESS score and PDSS score using the Pearson correlation, respectively (*p* < 0.01).

After that, we also conducted ANCOVA among three groups (voxel-level *p* < 0.01, cluster size >19 voxels, corresponding to a corrected *p* < 0.05 as determined by AlphaSim correction) and followed with a two-sample *post hoc t* test to explore the altered FC of the defined ROI between PD-EDS and PD-non-EDS subject groups (voxel-level *p* < 0.01, cluster size >3 voxels, corresponding to a corrected *p* < 0.01 as determined by AlphaSim correction). Besides, two sample *t* test was used to compare PD groups with healthy controls (the voxel-level *p* < 0.01, cluster size >18 voxels, corresponding to a corrected *p* < 0.01 as determined by AlphaSim correction). Age, gender, education, and gray matter volume were put into covariates as well.

## Results

### Demographic and Clinical Characteristics

Table 1 presents the clinical characteristics of all subjects. We found no significant differences in age and education level among the PD-EDS, PD-non-EDS, and HC groups. However, all PD-EDS subjects in our study were male, and did not match with the other two groups. The PD-EDS and PD-non-EDS had similar disease duration, H-Y staging, UPDRS-III scores, PIGD scores, tremor scores and medication. In addition, there were no significant differences in non-motor symptoms such as RBDSQ scores, MMSE scores, FAB scores, HAMD scores and HAMA scores (*p* > 0.05). As we expected, the PD-EDS patients showed higher ESS scores and PDSS scores relative to the PD-non-EDS patients (*p* < 0.05).

**TABLE 1:** Demographic and clinical characteristics of all subjects.

	PD-EDS	PD-non-EDS	HC	*P*
	(*n* = 17)	(*n* = 21)	(*n* = 19)	value
Gender (M/F)	17/0	12/9	8/11	< 0.001*
Age (y)	70.18 ± 6.66	66.05 ± 7.28	65.37 ± 4.67	0.058
Education duration (y)	12.06 ± 4.58	11.38 ± 3.20	11.68 ± 4.16	0.872
Disease duration (y)	5.26 ± 3.33	4.03 ± 3.25		0.256
H&Y staging	1.88 ± 0.49	1.90 ± 0.44		0.882
UPDRS-III score	26.35 ± 7.94	20.52 ± 11.06		0.076
PIGD score	6.76 ± 4.72	6.00 ± 4.54		0.615
Tremor score	4.53 ± 2.72	3.57 ± 2.80		0.295
Total LEDD (mg/d)	491.59 ± 247.04	359.00 ± 269.41		0.126
LEDD of DA (mg/d)	54.94 ± 46.40	42.33 ± 48.00		0.419
LEDD of LP (mg/d)	366.18 ± 198.42	240.48 ± 211.91		0.070
ESS score	11.53 ± 1.38	1.62 ± 1.07		< 0.001*
PDSS score	25.41 ± 14.79	14.86 ± 8.73		0.016*
RBDSQ score	3.00 ± 3.18	2.90 ± 2.88		0.923
MMSE score	27.53 ± 1.33	28.38 ± 1.36		0.060
FAB score	15.71 ± 1.57	15.67 ± 1.96		0.947
HAMD score	7.71 ± 6.78	5.57 ± 4.69		0.260
HAMA score	10.41 ± 6.76	8.57 ± 6.39		0.395

Data are presented as mean ± SD. PD-EDS, Parkinson’s disease with excessive daytime sleepiness; PD-non-EDS, Parkinson’s disease without excessive daytime sleepiness; HC, Healthy controls; M, Male; F, Female; y, Year; H&Y, Hoehn and Yahr; UPDRS, Unified Parkinson’s Disease Rating Scale; PIGD, Posterior instability and gait disturbance; LEDD, Levodopa equivalent daily dose; DA, Dopamine receptor agonists; LP, Levodopa preparations; ESS, Epworth Sleepiness Scale; PDSS, Parkinson’s disease sleep scale; RBDSQ, REM sleep behavior disorder screening questionnaire; MMSE, Mini Mental State Examination; FAB, Frontal Assessment Battery; HAMD, 17-item Hamilton Depression Rating Scale; HAMA, Hamilton Anxiety Rating Scale. *P < 0.05 was considered significant.

### ALFF Data

**FIGURE 1:**
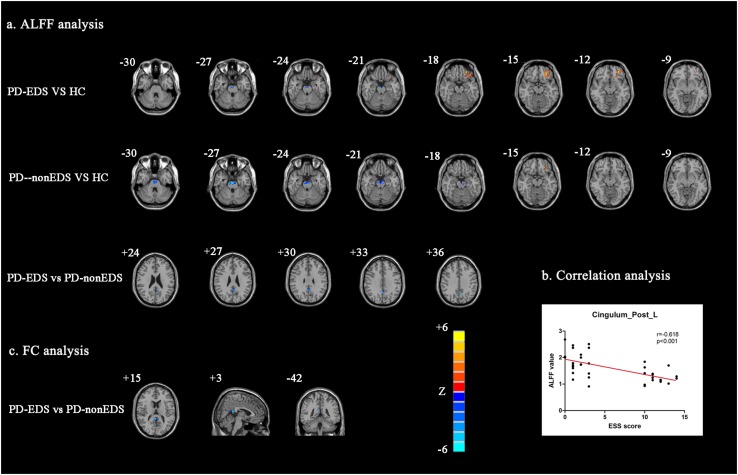
Analysis of ALFF and FC data **(a)**: Regions that showed significant ALFF differences via between-group comparisons (*P* < 0.01, AlphaSim corrected). **(b)** Correlation analysis between ESS scores and ALFF values of Cingulum_Post_L (*P* < 0.001). **(c)** Regions with which Cingulum_Post_L FC showed significant differences between PD-EDS and PD-non-EDS (*P* < 0.01, AlphaSim corrected). ALFF: The amplitude of low-frequency fluctuations; FC, Functional connectivity; PD-EDS, Parkinson disease with excessive daytime sleepiness; PD-non-EDS, Parkinson disease without excessive daytime sleepiness; HC, Healthy control subjects.

Among the three study groups, significant ALFF differences were found in Cingulum_Post_L, Pons, and Frontal_Mid_Orb_L. Next, the PD-EDS group showed decreased ALFF in Cingulum_Post_L compared with the PD-non-EDS group. Interestingly, compared with HC, both PD-EDS and PD-non-EDS study groups showed decreased ALFF in pons and increased ALFF in Frontal_Mid_Orb_L, and PD-non-EDS subjects were overall lower (Table 2 and Figure 1a).

**TABLE 2:** ALFF and FC analysis via between-groups comparisons.

Brain areas (AAL)	Cluster	MINI			*Z*
	size	coordinate			value
		X	Y	Z	
**ALFF analysis**					
PD-EDS VS. PD-non-EDS					
Cingulum_Post_L	22	−3	−51	27	−4.141*
PD-EDS VS. HC					
Pons	14	3	−15	−27	−3.768*
Frontal_Mid_Orb_L	66	−30	45	−12	5.761*
PD-non-EDS VS. HC					
Pons	65	3	−15	−27	−5.065*
Frontal_Mid_Orb_L	7	−24	33	−15	3.499*
**FC analysis Cingulum_Post_L as the ROI**					
PD-EDS VS. PD-non-EDS					
Cingulum_Post_R, extending to Precuneus_R	14	3	−42	15	−4.231*
PD VS. HC					
Cerebelum_Crus1_L	43	−21	−81	−21	−3.522

*ANCOVA and *post hoc* two-sample *t*-tests with age, gender, education and gray matter volume as covariates were performed to examine between-groups differences of the ALFF and FC. ALFF, The amplitude of low-frequency fluctuations; FC, Functional connectivity; MNI, Montreal Neurological Institute; PD-EDS, Parkinson’s disease with excessive daytime sleepiness; PD-non-EDS, Parkinson’s disease without excessive daytime sleepiness; HC, Healthy controls. The ALFF results are displayed at *p* < 0.01 corrected by AlphaSim, cluster size >5 voxels; the FC results are displayed at *p* < 0.01 corrected by AlphaSim, cluster size >3 voxels.

Figure 1b showed significantly negative correlation between ALFF values of Cingulum_Post_L and ESS scores in PD patients (*r* = −0.618, *p* < 0.001), but not with PDSS scores.

### FC Data

Likewise, in performing the ANCOVA and two-sample *post hoc t* tests, the PD-EDS group showed decreased Cingulum_Post_L FC with Cingulum_Post_R and Precuneus_R as compared with the PD-non-EDS group (Table 2 and Figure 1c). Compared with healthy controls, PD subjects showed decreased FC between Cingulum_Post_L and Cerebelum_Crus1_L (Table 2).

## Discussion

In this study, we observed the association between EDS in PD and altered neural activity at two levels, with ALFF at the local level and FC at the network level. At first, the abnormal ALFF of PD-EDS compared to HC in the Pons and Frontal_Mid_Orb_L supported the hypothesis that the wake-promoting pathway deficits were associated with EDS in PD. Furthermore, reduced ALFF of the left posterior cingulate cortex (PCC) and its reduced FC with right PCC and right precuneus in PD-EDS relative to PD-non-EDS hinted at the abnormal default mode network (DMN, e.g., PCC, precuneus, inferior parietal lobe, medial prefrontal cortex) in PD-related EDS. It is worth noting that the PD-EDS groups in this study were all males, which were not matched with other groups. To minimize gender bias, gender was analyzed as a covariate in this study. Nevertheless, we should be cautious when interpreting neural activity changes of PD-EDS. The PD-EDS related neural activity changes in this study might only be applicable to male patients.

The Pons contain the wake-promoting neuronal populations, including the serotonergic neurons of dorsal raphe nucleus, the noradrenergic neurons of locus coeruleus, the cholinergic neurons of the pedunculopontine nucleus (PPN) (Koval’zon, 2011) and parafacial GABAergic/glycinergic neurons (Alam et al., 2018). In this regard, ascending projections from these main pontine wake-promoting neurons activate the thalamus, which in turn arouses the neurons in the cerebral cortex. In addition, the ascending reticular activating system exists in the reticular formation of the Pons. All these wake-promoting pathways contribute to the maintenance of wakefulness. Hence, reduced ALFF in the Pons in this study might result in sleep-wake state instability owing to disruption of wake-promoting pathways, eventually associated with manifestation of EDS in PD. Previously, Braak et al. (2003) proposed the hypothesis of ascending brain stem degeneration in early PD, involving some aforementioned non-dopaminergic brain stem nuclei, which could also propel our understanding of sleep dysfunction in PD.

Applying structural MRI with morphometry, local brain stem atrophy was found in connection with EDS in PD (Gama et al., 2010); however, another structural MRI using voxel-based morphometry showed extensive cortical and subcortical brain atrophy in PD-EDS (Kato et al., 2012). Moreover, an early SPECT study reported decreased regional cerebral blood flow of the cerebral cortex, but no significant differences in brain stem perfusion between PD-EDS and PD-non-EDS (Matsui et al., 2006a). The discrepancy in these results could be attributed to different neuroimaging approaches, analysis methods, or whether to control the confounding factors. Nevertheless, an analogous rs-fMRI study (Wen et al., 2016) applied regional homogeneity and FC analysis methods and the results were completely different from ours. It is attractive that they enrolled early drug naïve PD patients, but they did not use control subjects. We made the adjustment by recruiting healthy control subjects. Moreover, despite that our PD subjects were not drug naïve, the PD-EDS group and PD-non-EDS groups were well matched in drug usage and other possible EDS-related factors in our study. Thus, according to our results, we still speculated that EDS was associated with the impaired wake-promoting pathways in PD.

Notably, PD-EDS patients had worse nocturnal sleep problems as assessed by PDSS in our study, which was consistent with previous work (Zhu et al., 2016). However, PDSS scores in PD patients were not statistically correlated with left PCC ALFF values, which were significantly negatively correlated with ESS scores. Thus, the decreased left PCC ALFF was specific for PD-related EDS. Further, we found reduced left PCC FC with right PCC and adjacent right precuneus when PD-EDS were compared to PD-non-EDS. Interestingly, the precuneus/PCC is the key node in the DMN (Fransson and Marrelec, 2008); in other words, there is a strong interaction between the precuneus/PCC and the rest regions of the DMN. Therefore, neural activity reductions of the precuneus/PCC might lead to functional impairments of the DMN. Indeed, the DMN is responsible for a conscious awareness and introspective state in which people are awake and alert (Mak et al., 2017), and functional decoupling of the anterior and posterior DMN nodes could indicate the loss of conscious awareness during deep sleep (Tagliazucchi and Van Someren, 2017), which added support to the reduced FC of posterior DMN nodes occurring in ESD in the present study. This could be bolstered by a recent rs-fMRI study, which showed that daytime sleepiness in healthy subjects was associated with impaired FC in the DMN independent of age and brain structure (Ward et al., 2013). In addition, the PCC is considered to be responsible for arousal and attention and its interactions with other networks may be linked to conscious awareness (Leech and Sharp, 2014), supporting the association we found with EDS. However, converging evidence suggests that there are structural and functional abnormalities of the PCC and DMN in other neurological and psychiatric diseases, for instance, Alzheimer disease, attention deficit hyperactivity disorder, and mood disorders (Leech and Sharp, 2014; Mohan et al., 2016). Herein, the role of the DMN in PD-related EDS still requires further investigation in future work.

It is well known that the DMN is strongly related to internal cognitive modes (Buckner et al., 2008). Since decreased DMN connectivity is observed in amnestic mild cognitive impairment (Petrella et al., 2011) and Alzheimer disease dementia. DMN connectivity is also significantly decreased in subjects who are deeply asleep (Tagliazucchi and Van Someren, 2017), lightly sedated (Greicius et al., 2008), descending into sleep (Samann et al., 2011), as well as EDS (Ward et al., 2013). These results suggest that decreased DMN connectivity during the daytime may reflect a more “sleep-like” state in the brain. This may also indicate a neural mechanism by which EDS can result in cognitive impairment. In our study, although there was no statistically significant difference between PD-EDS group and PD-non-EDS group, the score of MMSE of PD-EDS group was lower than that of PD-non-EDS group. This implies an interesting relationship between cognitive impairment and EDS, thus further research is needed.

Several limitations are existed. First, the ESS is a subjective self-reported questionnaire. To promote the sensitivity of discriminating subjects with similar levels of sleepiness, we defined an ESS score of 10 or greater as PD-EDS and the ESS score of 3 or less as PD-non-EDS. Even so, the objective instruments such as the Multiple Sleep Latency Test and polysomnography should be applied in future work. Second, our study had a relatively small sample size and the gender was not well matched among the three subject groups. The reason why no female was found in the PD-EDS patients could be due to the inadequate sample size, but it could also be due to that male gender is indeed associated with higher ESS score (Zhu et al., 2016). We think that the female PD-EDS will be enrolled if the sample size was further enlarged. It is necessary to enlarge the sample size to prove the universality of the results in all genders, or to further analyze neural activity differences between male and female PD-EDS. Besides, to minimize bias, gender was analyzed as a covariate. Third, we interpreted the results cautiously owing to the rs-fMRI methodology *per se*, and the hypothesis that we proposed about the probable neural mechanism in PD related EDS should be verified by animal experiments.

## Conclusion

In conclusion, our rs-fMRI study hypothesized the probable mechanism of the wake-promoting pathways and the DMN dysfunction underlying the EDS in male PD patients. It improved the understanding of this issue.

## Data Availability Statement

The datasets generated for this article are available on request to the corresponding author, or directly at w15895820585@163.com.

## Ethics Statement

The studies involving human participants were reviewed and approved by the Ethics Committee of The First Affiliated Hospital of Nanjing Medical University. The patients/participants provided their written informed consent to participate in this study.

## Author Contributions

MW was responsible for imaging data acquisition. XW, JL, and YS were responsible for clinical data collection and assessing scales. XW conducted the data analysis and wrote the manuscript. KZ and YY were responsible for experiment guidance, supervision, and article modification.

## Conflict of Interest

The authors declare that the research was conducted in the absence of any commercial or financial relationships that could be construed as a potential conflict of interest.

## Footnotes

1 http://www.restfmri.net/forum/dparsf

2 http://www.fil.ion.ucl.ac.uk

3 http://restfmri.net/forum/REST

4 http://afni.nimh.nih.gov/pub/dist/doc/manual/AlphaSim.pdf
